# Cerebral Abscess following Mechanical Thrombectomy for Ischemic Stroke: Report of a Case and Review of Literature

**DOI:** 10.7759/cureus.2824

**Published:** 2018-06-18

**Authors:** Shishir K Rao, Owais Ahmad, Farzana Tariq, Kushak Suchdev, Sandeep Mittal, Wazim Mohamed

**Affiliations:** 1 Neurology, Wayne State University School of Medicine, Detroit, USA; 2 Neurosurgery, Wayne State University School of Medicine, Detroit, USA

**Keywords:** brain abscess, fusobacterium necrophorum, acute ischemic stroke, thrombectomy

## Abstract

Cerebral infections have been reported after endovascular interventions such as embolization and coiling. Such complications are extremely rare and only one other case has been reported in a patient who underwent an endovascular therapy for ischemic stroke. We report a 32-year-old woman, who presented to our hospital with headaches lasting four weeks after an endovascular intervention for ischemic stroke via mechanical thrombectomy. Further investigations revealed a cerebral abscess in the area of the infarct. She was effectively treated with antibiotics in combination with stereotactic drainage and was discharged after she made a good recovery. A review of literature on cerebral abscesses after minimally invasive procedures such as endovascular intervention was also done and is being presented in this paper. A cerebral abscess can occur rarely after endovascular interventions. A high degree of suspicion is important in identifying patients with an abscess and appropriate treatment can prevent significant morbidity or even death.

## Introduction

Ischemic strokes involving a large vessel territory are potentially devastating causing significant physical disability and even death. While recombinant intravenous tissue plasminogen activator (rTPA) has shown to be effective in patients with ischemic strokes, its efficacy is limited in patients with large artery occlusion. Recently, multiple studies have been published revealing good outcomes in patients with ischemic stroke due to large artery occlusions, treated with endovascular therapies [1].

The risk of bacteremia following an endovascular procedure increases with the duration of procedure and can be as high as 32% in procedures lasting more than two hours [2]. Even with a high incidence of bacteremia, the risk of acquiring a clinical infection is low and these infections are mostly localized to the femoral artery [3].

Cerebral abscesses are rare yet challenging clinical problem with an incidence of 0.4 to 0.9 cases per 100,000 population. Intracerebral abscesses following embolization procedures have been previously reported [2]. However, there is only one report of an abscess developing after intra-arterial thrombolytic therapy for acute ischemic stroke [3]. To our knowledge, our patient is the first reported case of a cerebral abscess following mechanical thrombectomy. 

## Case presentation

**Figure 1:**
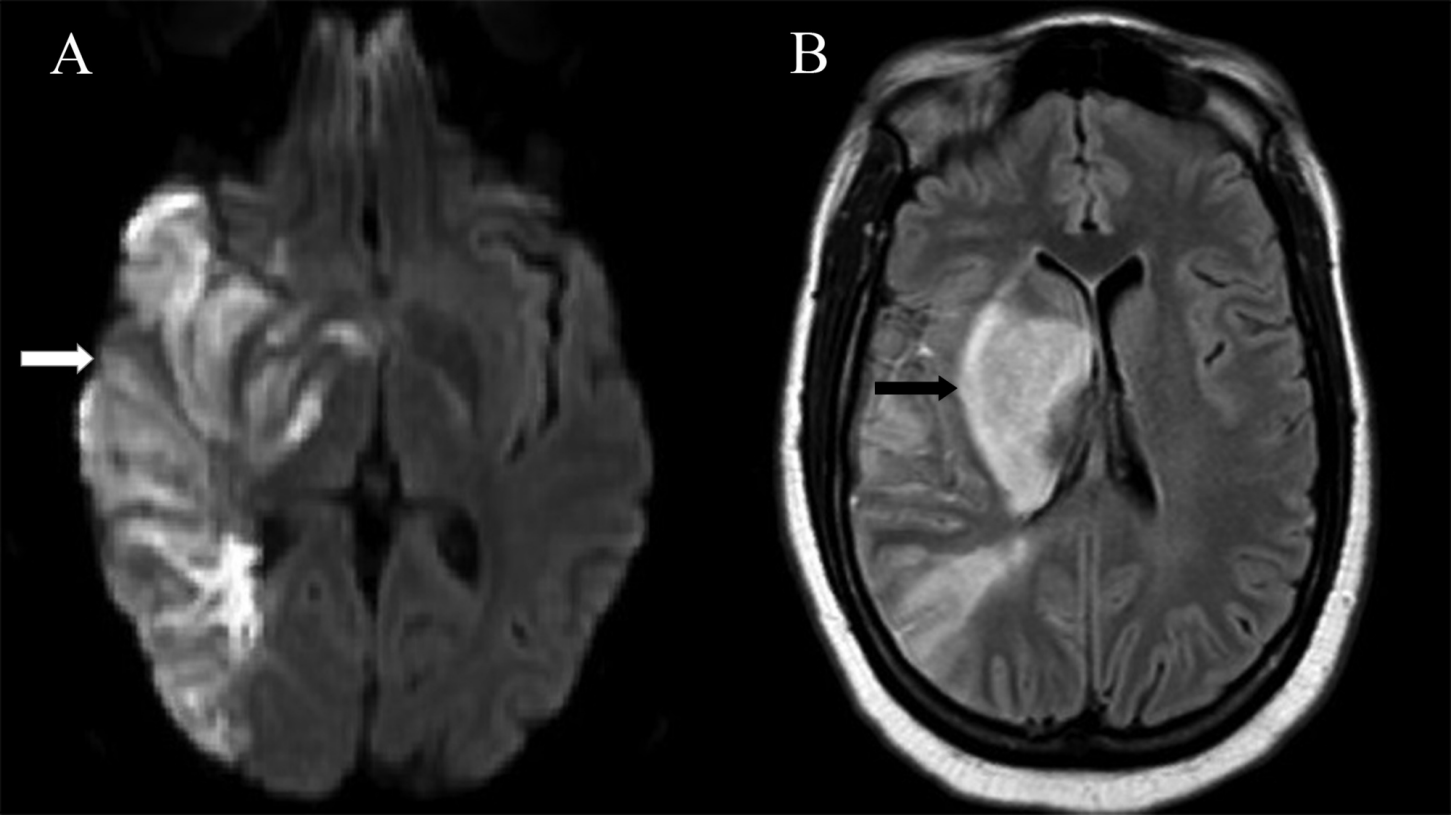
Initial MRI after ischemic stroke A: Axial diffusion weighted imaging (DWI) demonstrating diffusion restriction in the right temporo-parietal lobe (white arrow). B: Axial fluid attenuated inverse recovery (FLAIR) sequence with hyperintensity seen in the area of infarct in the right basal ganglia (black arrow).

A 32 year-old woman with a history of hypothyroidism and pre-eclampsia initially presented to an outside hospital with acute onset dense left hemiplegia, right gaze preference, and left-sided neglect. Her initial National Institute of Health Stroke Scale (NIHSS) was 14 and she had an admission Glasgow Coma Scale (GCS) of 10. A computed tomography (CT) angiogram of her neck revealed complete occlusion of the right cervical internal carotid artery (ICA). She was outside the time window for intravenous thrombolysis; however, she underwent mechanical thrombectomy using a stent retreiver device and aspiration (Penumbra System®, Alameda, CA). Immediately after the procedure, there was a successful restoration of the blood flow to the distal ICA, proximal middle cerebral artery (MCA), and to the anterior cerebral artery (ACA), with residual distal M2 occlusion. She was intubated for the procedure and was extubated in the following days. Her left-sided weakness persisted and a repeat CTA showed re-occlusion of the right cervical ICA. No further intervention was done and she was treated with aspirin and statin for secondary stroke prophylaxis. The stroke was deemed cryptogenic after work-up for a potential source was negative including an echocardiogram which demonstrated a normal ejection fraction, normal left atrial size, and negative bubble study. A workup for prothrombotic and hypercoagulable states were negative as well. Magnetic resonance imaging (MRI) of the brain was done which showed a large area of diffusion restriction with corresponding decreased apparent diffusion coefficient (ADC) and T2 hyperintensity in the right frontal, parietal, temporal lobes and in the basal ganglia with areas of hypointensities on gradient echo sequencing, which suggested infarction in these areas with some areas of hemorrhagic conversion (Figure 1). 

**Figure 2:**
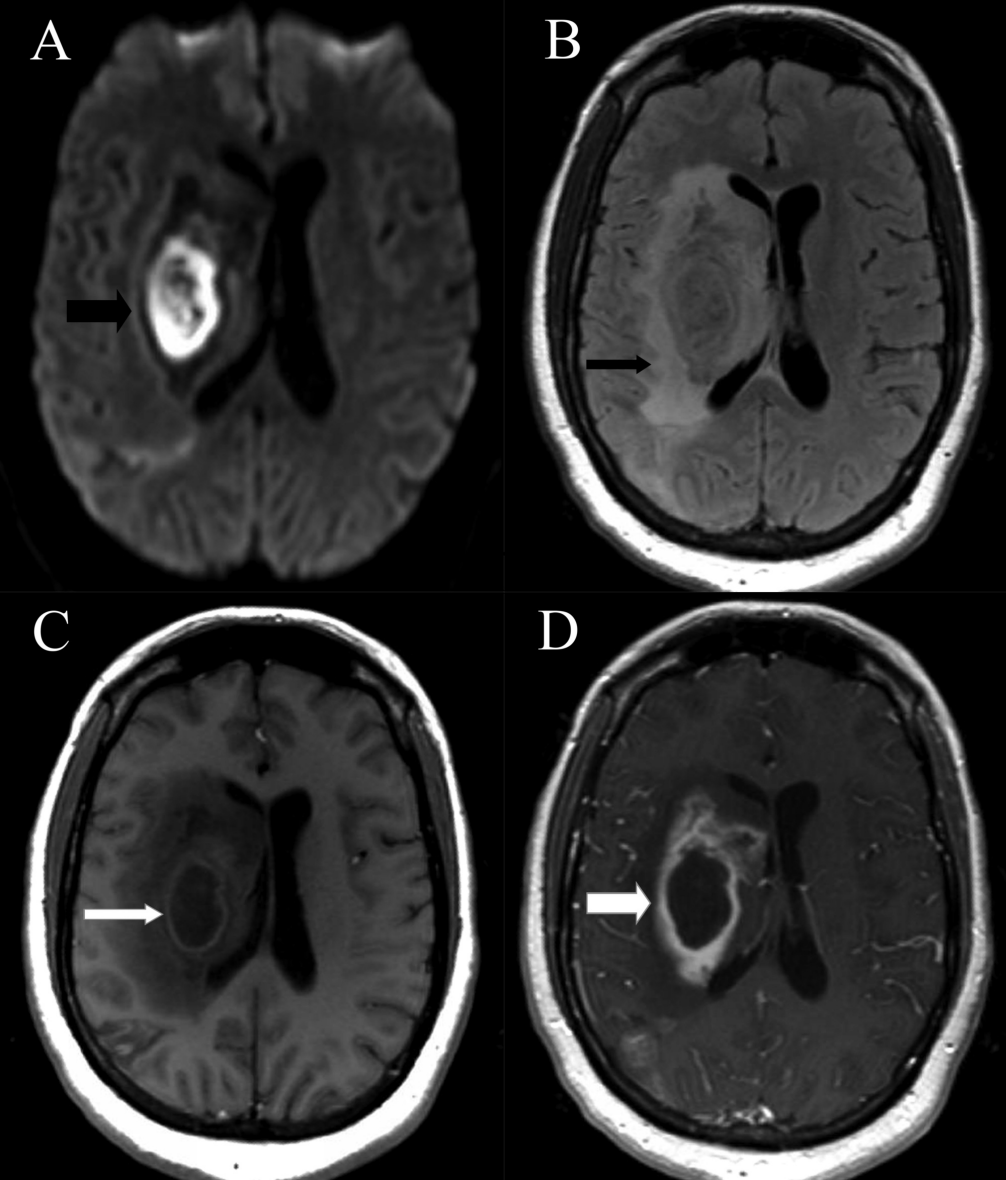
MRI brain after the onset of headache (Axial images) A: DWI sequence with restricted diffusion within the lesion (thick black arrow). B: FLAIR sequence with extensive vasogenic edema around the lesion (thin black arrow). C: T1 hypointensity in the basal ganglia with a thin surrounding wall (thin white arrow). D: T1 post-contrast image with a smooth thin ring enhancement of the lesion (thick white arrow).

Subsequently, she was discharged to an inpatient rehabilitation center. While at the rehabilitation center, about four weeks after her stroke, she developed moderate to severe insidious onset headache. A repeat MRI, done four days after the onset of headache, showed diffusion restriction (with corresponding decreased ADC) and a ring-enhancing lesion in the right basal ganglia which involved part of the previous ischemic stroke. An extensive area of T2 hyperintensity was seen around this lesion consistent with vasogenic edema (Figure 2). 

**Figure 3:**
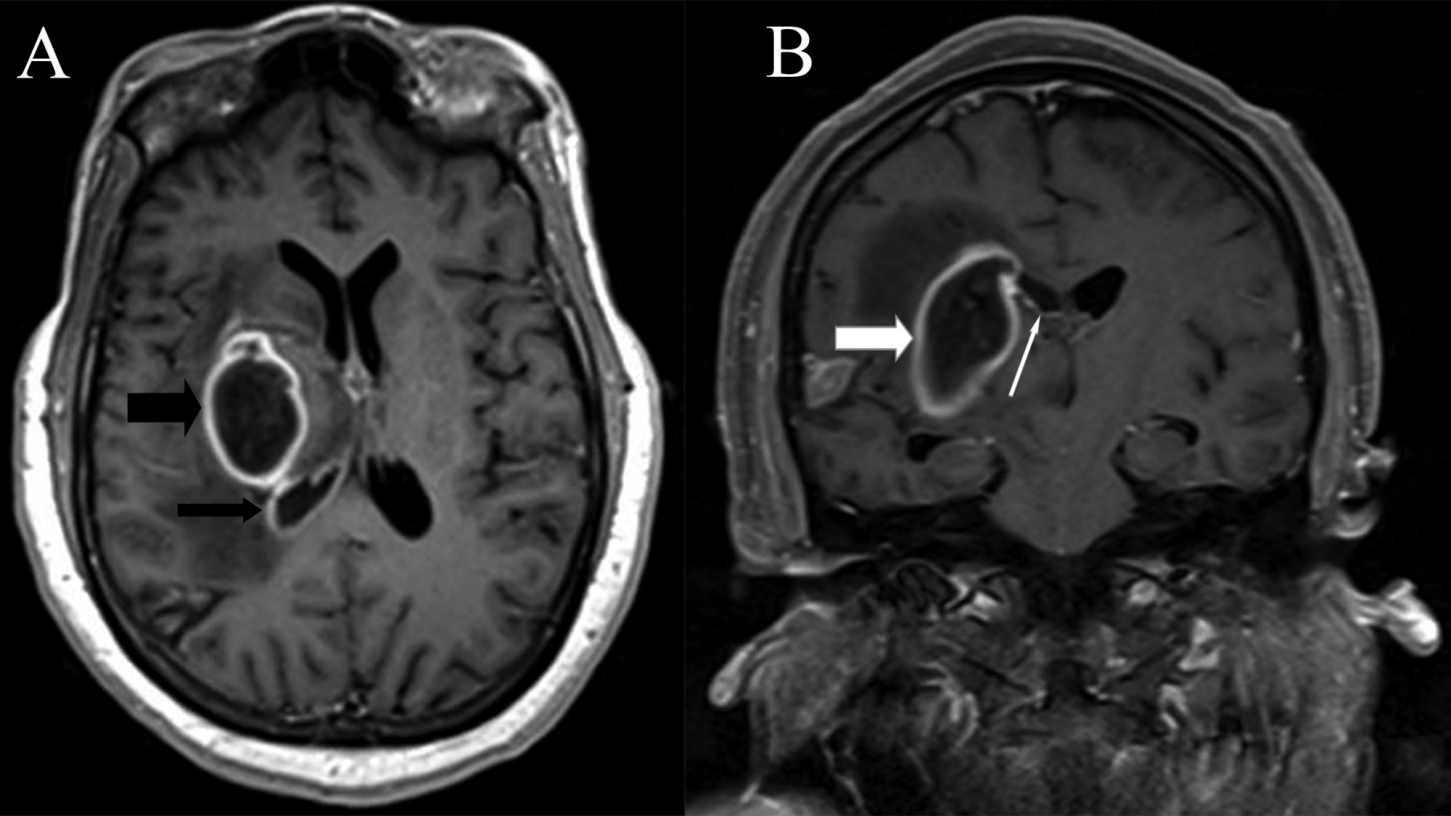
Repeat MRI after clinical deterioration A: Post-contrast T1 axial image demonstrating enhancement of the wall of the right lateral ventricle (thin black arrow) along with the previously described ring-enhancing lesion (thick black arrow). B: T1 post-contrast coronal section with ring-enhancing lesion (thick white arrow) and enhancement of the right ventricular wall (thin white arrow).

With a recent invasive procedure along with the MRI findings, the possibility of an abscess was entertained, even though she did not have systemic signs of an infection (afebrile, WBC count 7800/mm3, negative blood cultures). She was empirically started on broad-spectrum antibiotics (vancomycin, cefepime, and metronidazole) and admitted to our institute for further management. On day three of admission to our hospital, she developed a high-grade fever and had an acute deterioration in her mentation that progressed to coma. An MRI was repeated to evaluate for any progression of the disease and to obtain stereotactic images for drainage. In addition to the previously mentioned ring-enhancing lesion, the post-contrast sequences now demonstrated enhancement of the right lateral ventricular wall which was suggestive of ventriculitis (Figure 3).

She underwent a stereotactic drainage of the lesion, which aspirated purulent material. The patient was continued on broad-spectrum antibiotics. Vancomycin was discontinued after 10 days. Cefepime was switched to ceftriaxone, which along with metronidazole, was continued for a total of six weeks. An extensive laboratory workup was done which did not reveal a potential source of infection or immunocompromised state. Due to the high suspicion for an abscess and the purulent aspirate, a bacterial DNA probe was carried out on the aspirate, which revealed the presence of Fusobacterium necrophorum. Since Fusobacterium necrophorum is the implicated organism in Lemierre's syndrome, a surveillance of signs were carried out on the patient but failed to reveal neck pain or thrombosis of the internal jugular vein (imaged with an ultrasound of the neck). On post-drainage day one, her mental status improved and she progressed to her baseline prior to her discharge from the hospital.

## Discussion

The recent plethora of successful endovascular trials for acute ischemic strokes have paved the way for a new era in stroke therapies [1]. While endovascular treatment has remarkable advantages, there may be complications from the procedure including bacteremia and infections. There are a few case reports of cerebral abscesses following such procedures, but no formal study has been done due to the relatively low incidence of these complications [3-4] Even though the incidence of cerebral abscesses is low, it can be challenging to diagnose and treat them effectively.

It is well-known that ischemia or infarction of the central nervous system can cause degeneration of the blood-brain barrier (BBB), and loss in its integrity [3]. Recent studies have evaluated the role of tPA leading to destruction of BBB via activation of plasmin [5]. Endovascular therapy in the setting of ischemic stroke is associated with a further increased risk of disruption of BBB. The loss of the BBB makes the affected tissue much more vulnerable to hematogenous seeding or direct invasion in case of introduction of a contaminated foreign body during a procedure. The mechanical devices used for thrombectomy can also cause direct vascular injury leading to seeding [6].

Concomitant bacteremia in patients with a loss of BBB integrity can result in the spread of the infection to the central nervous system [7]. Most of the abscesses occur as a result of systemic infections. In our patient as well, the abscess occurred in the area of her recent infarct.

In the review of literature (PubMed search terms: brain abscess, cerebral abscess, stroke, thrombectomy, endovascular therapy) we found several case reports describing the formation of brain abscess after a stroke, which are summarized in Table 1. 

**Table 1:** Previously reported cerebral abscesses after stroke

Study	Age-Sex	Type of Stroke	Initial Treatment	Time to Abscess	Symptoms	Management	Micro-organism
Wang et al, 2015 [8]	58M, 42F	Pt 1: Ischemic—right hemispheric Pt 2: Ischemic—R MCA	Decompressive Craniectomy (both patients)	6 wks, 15 wks	Pt 1: fevers. Pt 2: Incidental	Surgery and antibiotics both patients	Pt 1: culture negative Pt 2: Pantoea agglomerans, Bacillus macerans
Rigante et al, 2013 [9]	49M	Hemorrhage—Left parietal	Medical Management	2 wks	Fevers, worsening neurological deficits	Surgery and antibiotics	Staph aureus
Yamanaka et al, 2011 [3]	75M	Ischemic—L MCA w/hemorrhagic conversion	tPA and mechanical thrombectomy	3 mos	None	Surgery and antibiotics	Staph epidermidis
Okami et al, 2011 [10]	51M	Thalamic Hemorrhage	Medical Management	3 mos	Worsening deficits	Surgery and antibiotics	Staph aureus
Thomas et al, 2009 [11]	57M	Hemorrhage (non-penetrating trauma)	Medical Management	4 wks	HA, hemiparesis	Surgery and antibiotics	E. coli
Kraemer et al, 2008 [12]	33F	Ischemic—L ACA/MCA w/hemorrhagic conversion	tPA and angioplasty	7 wks	Low-grade fever	Surgery and antibiotics	Group C. Streptococcus
Mason et al, 2007 [13]	80M	Thalamic ischemia	tPA	5 wks	Decline in mental status	Treatment Declined	Dematium-Fungus (autopsy findings)
Emmez et al, 2007 [14]	64M	Ischemic— L ICA	Medical management	10 wks	Decline in mental status	Antibiotics	Unknown
Sumioka et al, 1996 [15]		Putaminal Hemorrhage	Medical Management	2 mos	Fever	Surgery and antibiotics	Morganella morganii
Lee et al, 1994 [16]	64M	Basal Ganglia Hemorrhage-traumatic	Medical Management	4 wks	Fever, decline in mental status	Surgery and antibiotics	Pseudomonas aeruginosa
Chen et al, 1995 [7]	58F, 70M	Pt 1: Putaminal hemorrhage Pt 2: Ischemia- R MCA	Medical Management (both patients)	5wks, 5wks	Pt 1: Fever, HA Pt 2: Anisocoria	Surgery and antibiotics both pts	Pt 1: Klebsiella Pneumonia Pt 2: Culture negative
Kurihara et al, 1989 [17]	53M	Putaminal Hemorrhage	Medical Management	4 mos	Worsening deficits	Surgery and antibiotics	Staph aureus

The common finding in all of the above-mentioned cases is that the formation of brain abscess took place from two weeks to four months period after stroke. In most of these cases, patients presented with neurologic deterioration and/or headache. Most patients did not present with fever or other signs of sepsis. Seizure may also be a presenting symptom of brain abscess.

While CNS infections can be seen after infarctions, they have also been seen as a complication of other endovascular procedures. There are reports of brain abscesses after embolization of arteriovenous malformations, endovascular coiling of aneurysms and intra-arterial thrombolysis, however a cerebral abscess following mechanical thrombectomy and intra-arterial tPA administration for acute ischemic stroke has not been previously reported [2-3].

Fusobacterium has been most commonly associated with Lemierre’s syndrome and pharyngitis, but can also cause infections of gastrointestinal and urinary tract [18]. The organism is known to colonize the skin, thus resulting in sepsis and cerebral abscesses in patients with history of intravenous drug abuse or in patients with a central venous catheter such as the case in our patient [19]. Another risk factor for bacteremia and potential infection with Fusobacterium is the alteration in the pharyngeal environment caused by local infection along with invasive procedure such as endotracheal intubation [18]. Although endotracheal intubation is a benign and safe procedure, it has been noted that 12% of patients had positive cultures after the procedure [20]. As a remote possibility, since Fusobacterium is a normal commensal in the pharynx, the endotracheal intubation may have been the source for bacteremia, in our patient.

A high degree of suspicion is necessary to investigate patients with new-onset headache after a recent procedure which may have breached the BBB. Early identification of the cerebral abscess, treatment with appropriate antibiotics and evacuation are essential to decrease mortality. Antibiotic prophylaxis may be a reasonable option in select patients who are at risk for infection, such as those who are immunocompromised [4].

Systemic infections such as UTI, pneumonia, skin, and soft tissue infections, or transient bacteremia, likely increases the risk of developing a brain abscess after a stroke, and stroke patients are at increased risk for these systemic infections. It is therefore extremely important to minimize infectious sources, by eliminating unnecessary urinary catheterization, promoting aggressive pulmonary toilet, and vigilance in turning severely disabled patients to avoid decubitus ulcers.

## Conclusions

In summary, cerebral abscesses following mechanical thrombectomy for ischemic strokes are a rare entity, difficult to diagnose and require a high degree of suspicion. Patients with new onset headache after a cerebral infarction may require imaging to evaluate the underlying cause. These patients may present with subtle signs and not necessarily progress to sepsis. Early recognition and treatment of these patients is vital to improve outcomes.
